# Phospho-Tau and Chromatin Landscapes in Early and Late Alzheimer’s Disease

**DOI:** 10.3390/ijms221910283

**Published:** 2021-09-24

**Authors:** Laura Gil, Sandra A. Niño, Carmen Guerrero, María E. Jiménez-Capdeville

**Affiliations:** 1 Departamento de Genética, Escuela de Medicina, Universidad “Alfonso X el Sabio”, 28691 Madrid, Spain; lgilalb@uax.es; 2 Departamento de Bioquímica, Facultad de Medicina, Universidad Autónoma, de San Luis Potosí 78210, Mexico; saen08@gmail.com; 3 Banco de Cerebros (Biobanco), Hospital Universitario Fundación Alcorcón, Alcorcón, 28922 Madrid, Spain; CGuerrero@fhalcorcon.es

**Keywords:** cell nucleus, tau protein, aging, Alzheimer’s disease, chromatin architecture, nuclear lamin, epigenetic marks, histone code, heterochromatin, euchromatin

## Abstract

Cellular identity is determined through complex patterns of gene expression. Chromatin, the dynamic structure containing genetic information, is regulated through epigenetic modulators, mainly by the histone code. One of the main challenges for the cell is maintaining functionality and identity, despite the accumulation of DNA damage throughout the aging process. Replicative cells can remain in a senescent state or develop a malign cancer phenotype. In contrast, post-mitotic cells such as pyramidal neurons maintain extraordinary functionality despite advanced age, but they lose their identity. This review focuses on tau, a protein that protects DNA, organizes chromatin, and plays a crucial role in genomic stability. In contrast, tau cytosolic aggregates are considered hallmarks of Alzheimer´s disease (AD) and other neurodegenerative disorders called tauopathies. Here, we explain AD as a phenomenon of chromatin dysregulation directly involving the epigenetic histone code and a progressive destabilization of the tau–chromatin interaction, leading to the consequent dysregulation of gene expression. Although this destabilization could be lethal for post-mitotic neurons, tau protein mediates profound cellular transformations that allow for their temporal survival.

## 1. Introduction

Chromatin is a complex molecular framework of different nucleic acids and proteins that stabilize and ensure the functionality of the nuclear genome. This megastructure is contained inside a cell organelle, the nucleus, which consists of a protein scaffold called nuclear lamin (NL) that supports the nuclear matrix (NM). The long deoxyribonucleotide polymers are reversibly attached to the NM and the inner side of the NL in a tridimensional organization known as the interphasic nucleus.

The expression of specific genetic programs generates the identity of each cellular phenotype. Considering the 30,000 human genes, the task of selectively transcribing a fraction of these requires precisely conformed chromatin. Epigenetic modifications of chromatin components (such as histone methylations) and chromatin modifiers such as the tau protein modulate the organization of chromatin blocks and the access of specific sequences to transcription factories. This dynamic and plastic chromatin structure underlies the existence of over 200 different human cell types.

In this review, we analyze the epigenetic marks that define the aging process of human pyramidal neurons from the hippocampal CA1 region and the chromatin changes during their transition from senile to Alzheimer neurons. In addition, we delimit two chromatin landscapes associated with cytoplasmic or nuclear tau pathology observed in Alzheimer’s disease (AD).

## 2. Nucleosomes, Chromatin Types, and Nuclear Architecture

The chromatin fiber is the biologically functional DNA structure. The nucleosome consists of a negatively charged double DNA chain wrapped around a positively charged histone octamer built by pairs of H2A, H2B, H3, and H4 proteins. Nucleosomes are connected via 10–60 base pairs of linker DNA, forming a 10 nm chromatin fiber [1,2]. During interphase, the multiple nucleosome fibers fold into a local higher-order solenoidal 30 nm fiber. This structure, which constitutes the eukaryotic genome, is favored by the interaction of linker histone H1, cations such as Mg^2+^, polyamines, and other positively charged molecules. These molecules counterbalance the negatively charged fiber, allowing the compaction of nucleosomes in chromatin domains [3,4]. Furthermore, recent analyses employing Xray dispersion and cryo-electron microscopy identified supramolecular structures called “oligomers”. These 10 nm structures are formed by dynamic and disordered nucleosome fibers with intrinsic ability to self-assemble into large globules of chromatin stabilized by nucleosome-nucleosome interactions [2,5].

In vitro experiments employing synthetic nucleosomes and acetylated histones have shown globular condensates similar to liquid droplets in the presence of H1 and Mg. This exemplifies the nucleosomes’ remarkable auto-organization [6,7]. All modern technologies used to study chromatin, including cryo-electron microscopy, electron microscopy tomography, chromosome conformation capture (3C), and Hi-C [8,9,10,11], demonstrate that the interphasic chromatin is an irregular and dynamic structure with a fluid-like nature [12].

The sequence of DNA nitrogenated bases contains genetic information. Human genome sequencing makes it possible to classify DNA sequences transcribed and translated into proteins, non-coding DNA, and repetitive DNA sequences [13]. Repetitive DNA sequences account for more than 70% of the human genome [14]. Since different sequences derive from different chromatin structures, two functional states have been distinguished: euchromatin and heterochromatin [15]. Euchromatin or decondensed chromatin (10 nm fibers with nucleosome-free regions) is rich in GC bases and contains coding sequences of genes with high transcription levels and large chromatin movements [16,17]. Euchromatin sequences have characteristically hyperacetylated histone tails, which reduce electrostatic interactions with DNA and allow binding with transcription factors [18]. These histone modifications may also enable the formation of chromatin droplets [2]. The characterization of mono-, di-, and trimethylated residues in histone tails is emerging as a necessary code for the modulation of gene expression [19].

Condensed chromatin or heterochromatin is formed by 30 nm fibers or greater degree of packing with tight contacts between nucleosomes; it is rich in AT bases and poor in GC bases [20]. Heterochromatin mainly contains non-coding DNA sequences, especially repetitive sequences such as α, β, γ, I, II, and II satellite sequences; micro-, mini-, and macro-satellites; and diverse transposon types [13]. Constitutive heterochromatin has clear structural functions and scarce genetic density, while facultative heterochromatin adopts different conformations depending on its regulatory functions. Constitutive heterochromatin is mostly localized in telomeric and sub-telomeric regions, centromeric and pericentromeric regions, and in the satellites of the chromatin fibers [21]. It is characterized by a high condensation; hypoacetylated sequences enriched in repressive marks such as H3K9me3 and H4K20me3; and interaction with several proteins, such as HP1α/β, cohesins, and other chromatin proteins [19,22]. Although constitutive heterochromatin is considered functionally inactive, humans and other organisms show the transcription of centromeric and pericentromeric satellites through RNA polymerase II [23,24]. Transcripts of non-coding RNA have been reported under conditions of cellular stress, senescence, and during epithelial transition, both in embryonic development and malign transformation [25,26,27]. In contrast, facultative heterochromatin is more heterogeneous because it can adopt different conformations between 10 and 30 nm according to different conditions, such as transcriptional requirements (embryonic development genes), space (interchange between center and nuclear periphery, according to cellular activity), or genomic imprinting (monoallelic gene expression) [28]. Human facultative heterochromatin is hypoacetylated; its main epigenetic mark is H3K27me2/3, although it may present similar repressive marks to those of constitutive heterochromatin, such as H3K9me2/3 and H4K20me2/3 [19,29].

The scarce mobility of constitutive heterochromatin sequences leads to distinct localizations in the interphasic nuclei [30,31]. Lamin-associated domains (LADs) are genomic regions associated with the nuclear periphery that interact with the nuclear lamina (NL) [32]. As a consequence, euchromatic sequences rich in gene expression are preferentially localized in the center of the nucleus [33]. Collectively, heterochromatin plays a crucial role in the architecture of the interphasic nucleus and global chromatin stability. Last-generation techniques made it possible to observe the interphasic nuclei of eukaryotic cells, where large-scale structures with a diameter of approximately 200 nm build topologically associated chromatin domains (TADs) [34]. The proper structure and function of TADs require the docking of the CTCF protein and the action of cohesins. These domains restrict the regulatory landscapes where functional interactions between genes and regulatory elements occur [35]. In general, two epigenetic compartments are differentiated in the human and murine genome: compartment A and compartment B. Compartment A contains transcriptionally active chromatin that carries coding sequences; compartment B contains inactive chromatin and silenced repetitive intergenic sequences [34,36].

Finally, at the whole diploid nuclear level, the 46 chromosomic fibers are organized into territories (CTs) with each one having a specific localization [37]. The placement of these CTs is determinant for each cellular phenotype and is closely related to its genomic function and the global regulation of gene expression [38,39]. A recent live image analysis of completely differentiated *Drosophila* larvae showed a contrasting vision of chromatin topology. When living cells are studied without fixation, both chromatin types, active and repressed, are located at the nuclear periphery, close to the NL, and separated from the inner nucleoplasm. This creates a nuclear region devoid of central chromatin [40].

## 3. Nuclear Tau Modifies the Chromatin Landscape

**Figure 1:**
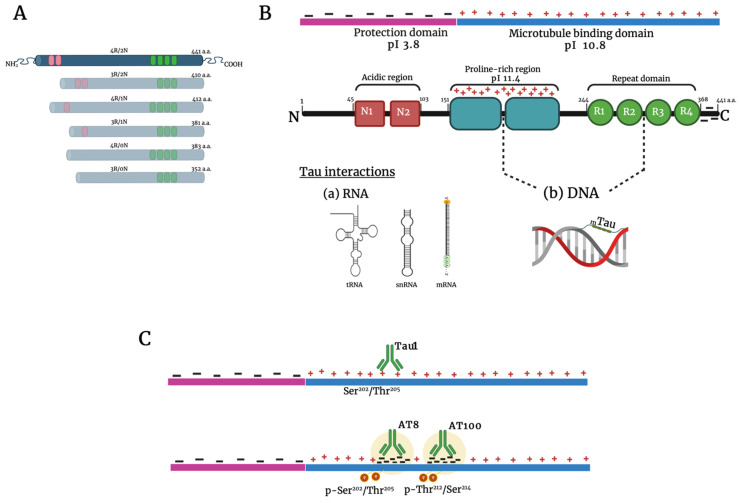
Tau protein structure and nuclear interactions. Structure of the six tau isoforms expressed in the adult human brain (**A**). Details of the longest isoform. It contains three major domains: an acidic N-terminal part (red), a proline-rich region (blue), and the basic microtubule-binding domain (green). The electrostatic interactions between domains allow the formation of condensed liquid tau droplets. The dotted line indicates the region where tau interacts with DNA and RNA (**B**). Upper line: Tau1 antibody recognizes the Ser202/Thr205 site. Lower line: The antibodies AT8 and AT100 bind with the phosphorylated sites Ser202/Thr205and Thr212/Ser214, respectively. Notice the change in the charge of the protein upon phosphorylation (**C**). Abbreviations: pI, regional isoelectric points; a.a., amino acids. Created with BioRender.com.

Tau is a soluble and highly flexible protein with multiple nuclear and cytoplasmic functions. Due to its intrinsically disordered protein (IDP) nature, tau barely displays a tertiary structure [41,42,43]. Tau is present in six isoforms because of alternative splicing (Figure 1A) [44]. The longest isoform, 2N4R, has a heterogeneous charge distribution [45,46]. The first 150 amino acids at the N-terminus, known as the protection domain (PD), have a negative charge. In contrast, the proline-rich domain (PRD, 151–243 aa) has numerous positive charges (Figure 1B). The C-terminus contains a basic microtubule-binding domain (MTBD), which is predominantly positively charged, followed by a negatively charged terminal region. The electrostatic interactions between domains and tau’s IDP nature allow the formation of condensed liquid tau droplets, both in vitro and in vivo [47,48,49,50,51]. Tau interacts with nuclear components such as nucleoli and nuclear speckles, as well as with cytoplasmic bodies such as stress granules and P granules [52,53,54,55,56,57].

Tau binds directly to nucleic acids. This has been demonstrated in vitro, in neuronal and non-neuronal cultures, and in the mouse brain. In addition, tau interacts with transference RNA (tRNAs) [58], small nuclear RNAs (snRNAs), small nucleolar RNAs (snoRNAs), and messenger RNAs (mRNAs). Through these bindings, tau reversibly condenses into one micrometer droplets, resulting in nuclear and cytoplasmic aggregates [57,58,59,60]. Tau also interacts with single and double DNA chains, building protein–DNA complexes [60,61,62]. The union site is the minor groove of the double strand, through the second half of the PRD and the R2 of the MTRD, giving rise to a “beads on a string” structure, like the histones in nucleosomes [63,64,65]. Depending on the tau/DNA ratio, other stable protein–DNA complexes may arise [66].

Chromatin-associated tau has been found in human and mouse brains [67]. Tau interacts with the whole nuclear DNA, as determined using chromatin immunoprecipitation followed by microarray hybridization (ChIP-on-chip) in primary embryonic neuron cultures. In euchromatin regions, tau binds with gene-coding and intergenic sequences, while in heterochromatin regions it interacts with repetitive sequences of long non-coding RNA rich in GC repeats [68]. GAGA sequences are uniformly distributed throughout the genome, but they are especially enriched in LADs. These sequences can reposition chromatin regions close to the nuclear envelope to silence genes [69]. In addition, a structural and functional relationship between tau and AT-rich alpha satellite repetitive sequences is involved in the organization of the centromeric heterochromatin ring from the nucleolus-associated domains (NADs) [53,70,71,72]. This partly explains the early observation of tau in the nucleolus. The nucleolus emerges around repetitive coding sequences from the active loci of ribosomal DNA (rDNA) or fibrillary centers (FCs) surrounded by repetitive centromeric DNA sequences included in the NADs [32,73]. These perinucleolar regions are enriched in repetitive elements, inactive X chromosome regions (Xi), and genes such as 5S rRNA, immunoglobulins, olfactory receptors, and zinc-finger proteins [74]. Furthermore, these regions are the site for pre-RNA processing and the assembly of pre-ribosome subunits [75,76].

In this respect, tau has been found both in regions of DAPI-positive heterochromatin [77,78,79] and DAPI-negative euchromatin from the nucleoplasm and nucleolus [52,78,80,81]. Gil and collaborators (2017) demonstrated the interaction of phosphorylated tau (p-tau) with global chromatin in pyramidal and granular neurons, as well as in epithelial cells of the colorectal mucosa and breast acinar cells [77]. The staining of p-tau was found in small dense foci within the chromatin distributed in the nucleus. In some cases, there was also staining in the NADs of pyramidal neurons and the LADs of epithelial cells, accompanied by a weak and diffuse nucleoplasm signal. Given that nucleosomes auto-organize in globular condensates like liquid droplets [6,7], interphasic chromatin has a fluid and irregular nature [12], and interphasic chromatin is compartmentalized [34], small p-tau foci might correspond with cell-type-specific tau-heterochromatin concentrates with significant elastic rigidity [38,39]. By contrast, faint nucleoplasm staining would be associated with the interaction between p-tau and euchromatin (compartment A) [82].

Chromatin is a dynamic structure; its topological changes determine cellular identity through specific patterns of gene expression, mainly through the intergenic and repetitive sequences organized in heterochromatin blocks (LADs and NADs) and nucleoplasmic heterochromatin. Tau is directly and indirectly related to the global heterochromatin, including the LADs and NADs [53,70,77,79]. Histone modifications are instrumental for chromatin dynamics, and recent data indicate that Tau could act as a chromatin modifier and be a new player in nuclear architecture due to its action on chromatin. Therefore, tau could also be a dynamic regulator of gene expression [83].

## 4. Epigenetic Histone Code, Nuclear Tau, and Chromatin Aging

The accumulation of DNA damage, mostly due to increased oxidative stress, represents a critical aging factor [84,85,86]. Post-mitotic neurons are especially vulnerable to oxidative stress because of their high oxygen requirements. They are also the longest living cells in the human body, and they cannot be replaced in the event of irreversible damage [87,88]. Different brain regions (cortex, hippocampus, cerebellum) and different neuron types (pyramidal or granular) within the same region display a wide range of age-related accumulation of DNA damage [89,90,91]. The diverse sensitivity to oxidative stress implies that the brain ages in a cell-type-specific manner [92]. Other well-documented consequences of DNA damage are the modification of the chromatin architecture, its connectivity, TADs organization, and epigenetic dysregulation [93]. The main epigenetic alterations associated with aged chromatin are limited accessibility due to histone loss, global decrease of constitutive heterochromatin, post-translational modifications of histones and DNA, disturbed miRNAs expression, and the replacement of LADs [94,95,96,97,98,99,100,101,102,103]. Altogether, these changes lead to the expression of silenced genes and repetitive DNA sequences, resulting in aberrant gene expression patterns [104,105,106,107,108]. Concerning the global heterochromatin loss, the histone methylation pattern in aging is characterized by an increase of transcriptionally active marks (H3K4me2-3, H4K16ac), a decrease of transcriptionally repressive marks (H3K9me2-3; H3K27me3) [94,109,110], and a dysregulation of H4K12 and H3K56 acetylation [111]. The downregulation of H3K36me, which also occurs during aging, is associated with aberrant gene expression and limits life span [112,113]. In addition, the trimethylation of H4K20 also increases with age in some tissues [114]. These patterns of epigenomic aging have only been characterized in replicative senescent cells, but data are scarce concerning post-mitotic neurons. Hernandez-Ortega and collaborators [115] reported high levels of H3K9me2 and H4K12ac in the pyramidal CA1 neurons of 18 senile subjects without neurological dysfunction. This result is interesting because low levels of H4K12ac are associated with memory impairment in the aged mouse brain [116] (Table 1).

It is widely accepted that tau protects DNA [117,118,119], preserves genome integrity [120,121,122,123], and is highly sensitive to cellular stressors [63,118,119], especially in neurons [79,115]. The absence of tau induces DNA double-strand breaks (DSBs), the phosphorylation of histone H2AX, and the disorganization of pericentromeric regions in mouse brains [70,119]. Conversely, its presence promotes a slow repair of hippocampal DSBs [119]. This protection could be associated with the progressive age-related increase in tau in pyramidal and granular hippocampal neurons (Table 1) [67,77].

Recent findings suggest a pathway whereby tau strengthens genome protection during aging. The function of tau is determined by its post-translational modifications, notably the phosphorylation of 80 serine or threonine residues distributed along the longest isoform [124,125]. Site-specific tau phosphorylation regulates its cytoplasmic and nuclear functions. Accordingly, two of the most relevant modulators of tau function are the sites recognized by the antibodies AT100 and AT8, located in Thr212 and Ser214, and in Ser202 and Thr205, respectively (Figure 1C) [126,127]. Pyramidal post-mitotic neurons from senile subjects have a very high immunopositivity to AT100, indicating abundant p-tau uniformly distributed within the nucleus, concomitant with high levels of H4K16ac [78,111]. This epigenetic modification inhibits the assembly of the 30 nm chromatin fiber, characteristic of heterochromatin regions [128], and it coexists with a significant aging-associated loss of histones [94,129]. It is tempting to hypothesize that a high tau concentration (around 100 µM) would be required to bind DNA, substituting the lost histones [65]. This binding would form nuclear gel-like p-tau droplets [50,51] where tau interacts with the positive charges [130] to keep the global heterochromatin condensed. Hence, centromeres, telomeres, and transposable elements would be stabilized [108].

The AT8 antibody recognition site of p-tau is an emblematic signature for AD diagnosis with a specific cytoplasmic location [131]. Nevertheless, pyramidal senile neurons clearly present AT8 immunopositivity in the nucleus interacting with the global chromatin, specifically with the transcriptionally inactive NADs [79]. This phosphorylation (sSer202/Thr205, AT8) may result in a change from the previous p-tau conformation (site Thr212/Ser214, AT100) that significantly improves its capacity for droplet organization and chromatin condensation [132,133]. It is remarkable that while AT100 gradually increases its nuclear presence throughout life, AT8 is mostly absent from chromatin and only appears in aged brains. Given its localization, we can conclude that it is associated with the transcriptional silencing of inactive rDNA genes and satellite repeats alpha and beta [78,134], as well as nucleoplasmic regions of facultative heterochromatin [79,135].

AT100 and AT8 are also present in chromatin from senile granular neurons. As in pyramidal neurons, AT100 progressively accumulates with age, displaying a global positivity in the nucleoplasm with a more intense signal in the LADs [77]. The perinuclear localization of AT100 would imply a silencing of repetitive DNA sequences associated with genomic stabilization [134]. In addition, the higher presence of AT100 could be related to the replicative character of granular cells. These cells require a greater DNA repairing capacity because of the continuous turnover derived from their functions in learning and memory [136,137]. AT8 is also present in granular senile neurons, while in younger cells it is almost absent (manuscript in preparation). As previously mentioned, AT8 recognizes the phosphorylated epitope Ser202/Thr205 while the Tau1 antibody marks the same residues but in the non-phosphorylated form [125,138]. The presence of tau in the nucleus, and especially in the nucleolus, has been widely reported [52,118,125,139]. Furthermore, it has been demonstrated that tau interacts with repetitive centromeric sequences rich in H3K9me3 and HP1α, using immunoelectron microscopy and ChIP assays from murine embryonic neuronal cultures [70]. In addition, the phosphorylation of this epitope in granular and pyramidal neurons from senile brains shows a conformational change in Thr212/Ser214 [81], which is crucial for the chromatin aging and the gene expression of brain-aging-associated genes [140].

## 5. Different Chromatin Landscapes Associated to Early and Late Ad Stages

AD diagnosis is performed post-mortem 5 to 10 years after the onset of the disease. Diagnosis is based on the cytoplasmic pathology of toxic neurofibrillary tangles and the presence of extracellular β-amyloid aggregates in the intermediate and late AD stages [141]. Nevertheless, the early landscape of the disease is a nuclear pathology involving chromatin alterations [142,143,144]. Other neurodegenerative disorders also display pathologic characteristics associated with dynamic chromatin modifications [145,146,147].

Recent investigations highlight that the nature of AD is not exclusively linked to aging [148] but to an epigenetic dysregulation of chromatin directly involving the histone code [142] and p-tau exiting the neuronal nucleus [79,115]. The progressive decrease of the epigenetic marks H3K9me3 and H412Kac in CA1 [115,142] is accompanied by a significant increment of H3K9me3 in centromeres and non-coding intergenic and intragenic regions [149,150]. Moreover, H4K20me3 in NADs and LADs [79] are also increased, implying high chromatin condensation. The trimethylation marks in H3K9 and H4K20 indicating chromatin repression are accompanied by the assembly of 30 nm chromatin fibers allowed by the decreased acetylated form H4K16ac [128,151]. Interestingly, the H416ac level, which is directly implied in heterochromatin organization, progressively increases with age, in parallel with AT100. In contrast, both decrease throughout AD progression [77,142]. In synchrony with this closed chromatin landscape during early AD stages (stages I–II according to Braak and Braak) [152], the following epigenetic modifications have been reported: an increased expression of histone deacetylases (HDACs) [153,154], especially of HDAC2, in the hippocampus [155]; and the cytoplasmic misplacement of DNA methyltransferase 1, DNA polymerase II (Pol II), and H3K4me3 [156,157]. In addition, minimal differences in DNA methylation have been observed in cortical neurons at early AD stages, independently of age-associated changes [158]. AT100 transfer from the nucleus to the cytoplasm reflects a progressive uncoupling of tau from chromatin, leading to the consequent destabilization of repetitive DNA sequences that conform NADs, LADs, and mobile genome elements. Although these changes would trigger neuronal death, the AD pyramidal neuron undergoes a profound chromatin transformation that allows its temporal survival. These epigenetic transformations determine the early AD phenotype of pyramidal neurons (Table 1) and involve aging-associated genetic dysregulation and global transcriptional changes that transform an aged neuron into a degenerated one [159].

The starting point of AD is marked by the nuclear phosphorylation of the AT8 site and the exit of AT100 from the nucleus to the cytoplasm [79,115]. In contrast, the late stages of the disease are marked by the massive presence of neurons in CA1 bearing neurofibrillary tangles in the somatodendritic compartments, positive to AT8 and AT100 [160,161]. The course of this process includes not only the aberrant neuronal cytoplasm but also a second transformation of the degenerated chromatin landscape. Important works have reported globally decondensed chromatin in several models, including (i) AD models in mouse and *Drosophila* bearing classic tau cytoplasmic pathology [143,144], (ii) forebrain neurons derived from human pluripotent stem cells, and (iii) the prefrontal cortex of autopsied brains, where abundant neurofibrillary tangles were taken as clinical evidence of AD [142]. The disappearance of repressive marks such as H3K9me3 and H4K20me3 occurs in concert with increased histone acetylation, such as H2BK5ac, H3K14ac, H3K18ac, H3K23ac, H4K5ac, and H4K12ac [162]. Together with the cytoplasmic tau pathology, H3K9ac is placed both in compartment A and compartment B, confirming the decondensed state of global chromatin and the modification of nuclear architecture in late AD stages (stages V–VI, according to Braak and Braak) [79,143,152] (Table 1). Around 57,800 promotor sequences associated with H3K4me3 and 151,477 active potentiating sequences related to H3K27ac have been identified [163]. Additionally, H3K27ac is highly present in the human entorhinal cortex affected by tau pathology [164]. Analysis of the integral transcriptome of the human hippocampus in late AD stages revealed 2064 genes, 47 lncRNAs, and 4 miRNAs with dysregulated expression. Up-transcribed genes were associated with tau phosphorylation, neurogenesis, synaptic vesicle traffic, long-term potentiation (LTP), and neurite growth [165].

The depletion of global heterochromatin and the closed chromatin structure of repetitive DNA sequences implies the activation of transposons [166,167] and CpG nucleotides dispersed in repetitive DNA [168,169], as well as the destabilization of centromeric and pericentromeric DNA sequences from NADs and LADs [70,79]. Given that these processes manage the organization of chromatin spaces, their structure loss implies the destabilization of topologically associated chromatin domains and CTs [170] and the loss of the post-differentiated character of the neuronal nucleus [171].

Importantly, two processes occur simultaneously throughout the process of AD pathology: the genesis of the AD neuronal phenotype, and the progressive loss of pyramidal cells that reenter the aberrant cell cycle [172,173,174]. This means that two pyramidal neuronal populations with different degrees of degeneration coexist in the hippocampus of AD patients. The first population is post-mitotic neurons that exit the quiescent state by starting an aberrant cell cycle that eventually drives them to cell death. The second refers to neurons with AD phenotype, whose profound chromatin, nuclear, somatodendritic, and axonal transformation materialize the characteristic AD neurodegeneration [79,175]. Recent works in induced neurons from patient-derived fibroblasts have linked oxidative stress and DNA damage to aberrant cell cycle re-entry, apoptosis activation, and, importantly, the loss of a differentiation state and a “return to immaturity” [176,177]. The RNA-seq analysis of 82 temporal cortex neurons from a late AD sample identified the up-regulation of 600 genes related to epithelial–mesenchymal transition and cancer along with the downregulation of genes associated with mature neuronal identity [171].

## 6. Conclusions and Future Directions

The accumulation of oxidative stress, DNA damage, and other nuclear and cytoplasmic alterations drive aged cells to their biological limits. Once their duplicative potential is depleted, replicative cells can remain in a limbic senescent state. Although this process allows them to stop their eternal youth drive in most cases, it increases the risk for developing the malign cancer phenotype. In contrast, post-mitotic cells maintain extraordinary functionality despite advanced age, but they lose their identity and finally die. The hippocampus is the brain’s compass, and the progressive neuronal loss is alleviated through the genesis of a surviving cell, the AD neuron. This aberrant phenotype grants the AD brain a few more years of life until its final defeat. In this sense, nuclear tau plays a crucial role as an aging chromatin biomarker in AD and cancer [77,171].

Over the course of three decades, pharmacological treatments for AD patients have targeted β-amyloid and tau protein [178]. The objective has been to reduce and/or avoid the development and spreading of their toxic aggregates in the neuropile and neuronal cytoplasm. The disappointing results of therapies against β-amyloid shifted the focus toward neurofibrillary tangles of hyperphosphorylated tau protein [179]. Many small molecules have recently been designed to inhibit or block tau phosphorylation in order to reduce its cytoplasmic toxicity, which characterizes late AD [180,181,182]. Our results indicate that early disease stages are defined by a nuclear dysregulation associated with NL changes, increased nuclear amounts of p-tau, and tau transfer into the cytoplasm. Future research of p-tau inhibitors in AD animal models will enable the possibility of slowing down nuclear p-tau accumulation and delaying cytoplasmic pathology.

## Figures and Tables

**Table 1:** Summary of chromatin and tau modifications in human CA1 pyramidal neurons through aging and AD evolution. Two epigenetic landscapes are defined for AD, considering an early and a late stage.

Epigenetic Stages in Neurons
Aging: condensed global chromatin and nuclear tau
ON transcriptionally repressive marks (heterochromatin): H3K9me3
OFF transcriptionally active marks (euchromatin): H4K16ac
Dysregulated: H4K12ac
Nuclear tau: AT100 increase and expression of AT8
Early Alzheimer’s Diease: Condensed heterochromatin (LADs, NADs) and Lamin A
ON transcriptionally repressive marks (constitutive heterochromatin): H3K9me3, H4K20me3
ON hippocampal HDAC2 expression
OFF transcriptionally active marks (euchromatin): H4K16ac
Ectopic cytoplasmic localization: H3K4me3, DNA methyltransferase 1, RNA Pol II
Nuclear tau: fading
Late Alzheimer’s Diease: Decondensed global chromatin and cytoplasmic tau
ON global acetylation
OFF methylation (heterochromatin): H3K9me2, H4K20me3
ON H3K9ac (euchromatin and heterochromatin)
Cytosolic tau: Tangles
